# Clinician attitudes towards cancer treatment guidelines in Australia

**DOI:** 10.1186/s13104-023-06356-5

**Published:** 2023-05-16

**Authors:** Mia Bierbaum, Gaston Arnolda, Jeffrey Braithwaite, Frances Rapport

**Affiliations:** Australian Institute of Health Innovation, Macquarie University, Macquarie Park, NSW 2113 Australia

**Keywords:** Neoplasms, Radiation oncology, Medical oncology, Surgical oncology, Practice guidelines, Guideline adherence, Evidence based practice, Cancer

## Abstract

**Objectives:**

Clinical Practice Guidelines (CPGs) are designed to guide treatment decisions, yet adherence rates vary widely. To characterise perceived barriers and facilitators to cancer treatment CPG adherence in Australia, and estimate the frequency of previous qualitative research findings, a survey was distributed to Australian oncologists.

**Results:**

The sample is described and validated guideline attitude scores reported for different groups. Differences in mean CPG attitude scores across clinician subgroups and associations between frequency of CPG use and clinician characteristics were calculated; with 48 respondents there was limited statistical power to find differences. Younger oncologists (< 50 years) and clinicians participating in three or more Multidisciplinary Team Meetings were more likely to routinely or occasionally use CPGs. Perceived barriers and facilitators were identified. Thematic analysis was conducted on open-text responses. Results were integrated with previous interview findings and presented in a thematic, conceptual matrix. Most barriers and facilitators identified earlier were corroborated by survey results, with minor discordance. Identified barriers and facilitators require further exploration within a larger sample to assess their perceived impact on cancer treatment CPG adherence in Australia, as well as to inform future CPG implementation strategies. This research was Human Research Ethics Committee approved (2019/ETH11722 and 52019568810127, ID:5688).

**Supplementary Information:**

The online version contains supplementary material available at 10.1186/s13104-023-06356-5.

## Introduction

Clinical Practice Guidelines (CPGs) are designed both to guide treatment decision making through synthesis of the best available evidence, and to reduce unwarranted clinical variation [1]. Cancer treatment CPG-adherent care is associated with improved patient outcomes [2, 3]; however, non-adherence persists [4–15]. In order to explore perceived barriers and facilitators to cancer treatment CPG adherence in Australia, a mixed methods study [16] was conducted. Previously published results include: a review characterising perceived determinants of cancer treatment CPG adherence [14]; a review presenting cancer treatment CPG-adherence rates and associated factors in Australia [15]; and a qualitative study identifying perceived barriers and facilitators to cancer CPG adherence in New South Wales (NSW), Australia, mapped across five themes [17]. This manuscript represents the second empirical phase of this sequential study [16] that aimed to quantify and generalise previously identified qualitative findings [17] in a broader sample of oncologists across Australia. Survey results are presented and integrated with previous qualitative findings.

## Main text

### Survey methods

A purpose-designed survey was developed that was informed by interview findings from a previous study [17] and the literature [18–21]. The 13-question, self-administered survey assessed clinician attitudes and demographic details. One question was the previously validated tool ‘Attitudes towards clinical practice guidelines’ requiring a 6-point Likert scale response (strongly agree to strongly disagree) to 18 sub-questions [22, 23]. Approximately n = 200 completed surveys were anticipated [16].

### Three approaches to recruitment were used

1. In February 2020, senior oncologists from seven hospitals (across four geographical catchments, representing half of the population of NSW, Australia [24]) invited, via email, an unspecified number of hospital-based oncologists to complete the survey, sending a reminder in March 2020. The invitation included a survey pack (an online survey link, a participant information and consent form, and a gift-card draw entry-form). Purposive sampling by the senior oncologists ensured clinicians from a range of seniority and disciplines were invited [25]. Recruitment was paused because of concerns regarding coronavirus disease (COVID-19) pandemic-related clinician burnout [26] and recommenced in March 2021.
2. In May 2021, the Clinical Oncology Society of Australia (COSA) emailed invitations to participate, with a survey pack, to 257 oncology specialist Society members; reminders were sent in June 2021.
3. In June 2022, 290 hard copy invitations and surveys (along with a survey pack and reply-paid envelope) were posted to clinicians across the seven hospitals who were listed on a NSW Government website of oncology Multidisciplinary Team (MDT) members (“CANREFER” [27]). This excluded clinicians who had previously completed the survey. All participants were encouraged to forward the survey link to colleagues.

### Analysis

Descriptive statistics of the characteristics of the clinician sample were calculated using the Statistical Package for Social Scientists, version 21 (SPSS, Chicago, USA), and presented as counts and proportions. An attitude score was calculated using the Attitudes Regarding Practice Guidelines tool [22, 23]. An analysis of variance was conducted to assess the statistical significance of differences in mean scores across clinician subgroups (Supplementary File 1). The associations between frequency of referring to CPGs, clinician demographics and practice patterns was explored, with statistical significance assessed by Fisher’s exact test [28]. Thematic analysis was conducted to examine open-text responses [29].

Mixed method data integration of survey findings with previously reported interview findings was conducted at the methods level, through *building*, where interview findings informed the survey development [30]. The triangulation of the two data sets and data collection methods aimed to enhance trustworthiness [31, 32] and corroborate the interview findings through a larger sample [30]. The thematically coded interview data was previously *quantitised* [33] and integrated with the survey data at the data interpretation and presentation level [30] through a visual display of a thematic conceptual matrix [30, 34–36]. Each comparable finding from the interview and survey studies was identified and assigned an alphanumeric code, using the Methods for Aggregating The Reporting of Interventions in Complex Studies (MATRICS) method [35] (Interview findings represented by “A”, survey findings by “B”, each interview subtheme represented by a number). Survey findings were assessed as: (1) convergent with interview findings (in *agreement*); (2) as offering information on the same issue that was *complementary*; or (3) as contradictory (*discordant*) [35, 37]. Interview subthemes that weren’t assessed in the survey were labelled *silent (expected)* [37]. Findings were labelled *discordant*, *neutral* [38], or in *agreement*, if less than, exactly, or more than 50% of survey respondents reported the finding, respectively. Findings were merged into summary statements [35] and presented within the qualitative thematic framework [17].

### Survey Results

In total, 48 surveys were completed (19, 15 and 14 surveys returned in each wave respectively), yielding an estimated 5.8% and 4.8% response rate in second and third waves (with six postal surveys returned to the sender). An overall response rate was not calculated owing to snowball sampling and an unknown number of clinicians approached in wave one.

Most clinicians were aged 40–69 years and practicing in NSW. Most were medical oncologists (MOs; n = 15), radiation oncologist (ROs; n = 10) or surgeons (n = 15), practising as staff specialists or Visiting Medical Officers, who graduated from medicine between 1980 and 2009, completing their medical and oncology training in Australia. Clinicians practiced a mix of public and private practice and typically only practiced in metropolitan hospitals. Most clinicians were members of an MDT, attending 1–4 MDTs more than once per week. The most common cancers treated were breast, colorectal, upper gastrointestinal, lung, skin and haematological cancers. Just under half of the clinicians treated one cancer stream, while almost a third treated four or more cancer streams (Table 1). Most surgeons (11/15) and haematologists (3/3) reported treating one cancer stream, while most MOs (12/15) and ROs (8/10) reported treating multiple cancer streams.

**Table 1:** Participant demographic characteristics

Clinician demographics and practice characteristics		n	%
Age	< 40y	8	16.7%
	40-49y	16	33.3%
	50-59y	13	27.1%
	60y+	11	22.9%
State of practice	NSW	39	81.3%
	Other	9	18.8%
LHD of practice*	SESLHD	13	33.3%
	WSLHD	11	28.2%
	SWSLHD	8	20.5%
	Other	7	17.9%
Specialty	MO	15	31.3%
	Surgery	15	31.3%
	RO	10	20.8%
	Haematology	3	6.3%
	Other	5	10.4%
Position	Staff Specialist	31	64.6%
	Visiting Medical Officer	11	22.9%
	Other	6	12.5%
Year graduated from medicine	Since 2010	6	12.8%
	2000–2009	10	21.3%
	1990–1999	14	29.8%
	< 1989	17	36.1%
Year completed oncology specialty	Since 2010	14	30.4%
	2000–2009	16	34.8%
	1990–1999	8	17.4%
	< 1989	8	17.4%
Country graduated medicine in^^^	Australia	39	83.0%
	Other	8	17.0%
Country completed oncology specialty training in^^^	Australia	43	91.5%
	UK^#^	2	4.3%
	Combined Australia and UK/CA^##^	2	4.3%
Proportion of clinician practice spent in public practice	< 25%	8	16.7%
	25–49%	10	20.8%
	50–74%	8	16.7%
	75–100%	22	45.8%
Proportion of clinician practice spent in metropolitan areas	< 75%	3	6.3%
	75% or more	45	93.8%
Cancer streams clinicians worked in	Breast cancer	16	33.3%
	Colorectal cancer	13	27.1%
	Lung cancer	12	25.0%
	Melanoma	12	25.0%
	Haematological cancer	11	22.9%
	Skin cancer	11	22.9%
	Prostate cancer	9	18.8%
	Head and Neck cancer	9	18.8%
	Gynaecological cancer	9	18.8%
	Other cancer streams	25	52.1%
Number of cancer types treated	1	21	43.8%
	2–3 cancer streams	12	25.0%
	4–5 cancer streams	9	18.8%
	6 or more cancer streams	6	12.5%
Membership of MDTs^^^^	None	2	4.5%
	1 or 2 MDTs	20	45.5%
	3 or more MDTs	22	50.0%
Frequency of MDT attendance	More than once per week	28	60.9%
	Weekly or less often	18	39.1%
Frequency clinicians refer to CPGs	Routinely	17	37.0%
	Occasionally	16	34.8%
	Rarely	11	23.9%
	Never	2	4.3%
Frequency that practice is adherent to CPG recommendations	Routinely	32	69.6%
	Occasionally/More than half the time	14	30.4%

^*^Local Health Districts (LHDs); South Western Sydney LHD (SWSLHD), South Eastern Sydney LHD (SESLHD), Western Sydney LHD (WSLHD), ^^^One response missing, ^^^^Four responses missing; excluded from calculation of percentages. ^#^United Kingdom, ^##^Canada

Clinicians reported staying up-to-date by attending conferences (n = 39), reading journals (n = 36), attending MDTs (n = 23), discussing cases with colleagues (n = 15) and attending journal clubs or educational meetings (n = 12). Many clinicians routinely or occasionally referred to CPGs when making treatment decisions (33/46) and estimated that their practice was routinely adherent with CPG recommendations (32/46). Of clinicians who routinely referred to CPGs, most (13/17) treated two or more cancers, while those who referred to CPGs less frequently, typically only treated one cancer stream (17/29). Clinicians mainly reported using CPGs developed by: eviQ (n = 22), National Comprehensive Cancer Network (n = 22), American Society of Clinical Oncology (n = 15), Cancer Council Australia/National Health and Medical Research Council (n = 13) and the European Society of Medical Oncology (n = 12) CPGs. Clinicians referred to CPGs to ensure their practice was current and evidence based (n = 23), to support treatment plans for complex/rare/unfamiliar cases (n = 12), and/or to seek consensus opinion when evidence was lacking (n = 2). Clinicians reported not referring to CPGs when CPGs were out-of-date (n = 6), when clinicians felt they were well informed through MDTs and journal club (n = 5), and when CPGs were not locally relevant (n = 2) or too generic (n = 2).

The mean CPG attitude score was 42.6 (95%CI 40.4–44.8), ranging from 23 to 59, with 60 being the most positive score possible. No significant differences in mean scores were found across clinician subgroups (Supplementary File 1): average scores indicated a tendency for positive attitudes towards cancer CPGs. The only clinician characteristics that were significantly associated with frequency of referring to CPGs was the age of clinicians (p = 0.007) and number of MDTs clinicians attended (p = 0.03), with younger clinicians and those attending more MDTs referring to CPGs more frequently (Table 2). This may indicate that clinicians attending more MDTs (who treat more cancer sites), utilise CPGs to remain current with the evidence base across multiple cancer sites. Similarly, younger clinicians may engage with CPGs more frequently to support their professional development. Neither higher attitude scores, nor referring to CPGs necessarily result in CPG adherence, however, as demonstrated by the wide variation in rates across Australia and different cancer streams detected previously [15].

**Table 2:** Frequency of referring to CPGs by clinician characteristic

Clinician demographics and practice characteristics		Routinely/ occasionally	%	Rarely/ never	%	P value (FET)
Clinician age	20–49 years	21	91.3	2	8.7	0.007*
	50–79 years	12	52.2	11	47.8	
State of practice	NSW	28	73.7	10	26.3	0.67
	Other	5	62.5	3	37.5	
Specialty	Medical oncology	9	64.3	5	35.7	0.43
	Radiation oncology	9	90.0	1	10.0	
	Surgery	9	60.0	6	40.0	
	Haematology	3	100.0	0	0.0	
	Other	3	75.0	1	25.0	
Professional position	Staff specialist	24	80.0	6	20.0	0.17
	Visiting Medical Officer	6	54.5	5	45.5	
	Other	3	60.0	2	40.0	
Year graduated from medicine	< 1999	20	64.5	11	35.5	0.17
	> 2000	13	86.7	2	13.3	
Year completed oncology training	< 1999	8	53.3	7	46.7	0.09
	> 2000	24	80.0	6	20.0	
Country medical training completed in	Australia	27	71.1	11	28.9	1.0
	Other	6	75.0	2	25.0	
Country oncology training completed in	Australia	31	73.8	11	26.2	0.32
	UK	1	50.0	1	50.0	
	Australia and UK/Canada	1	50.0	1	50.0	
Proportion of clinical practice in public settings	Less than 50%	12	70.6	5	29.4	1.0
	50% or more	21	72.4	8	27.6	
Proportion of clinical practice in metropolitan settings	< 75%	1	33.3	2	66.7	0.19
	75% or more	32	74.4	11	25.6	
Number of cancer streams clinician treats	1	13	61.9	8	38.1	0.38
	2–3	9	75.0	3	25.0	
	4 or more	11	84.6	2	15.4	
Membership of MDTs	No	1	100.0	0	0.0	1.0
	Yes	32	71.1	13	28.9	
	1 or 2 MDTs	11	57.9	8	42.1	0.03*
	3 or more MDTs	20	90.9	2	9.1	
Frequency of MDT attendance	More than once per week	22	78.6	6	21.4	0.19
	Weekly or less often	10	58.8	7	41.2	
Frequency clinicians’ practice is adherent with CPG	Routinely	24	75.0	8	25.0	0.49
	Occasionally / More than half the time	9	64.3	5	35.7	

Significance at p = 0.05

### Integration with previously published interview findings

Comparable findings from this survey and the previous interview study [17] were integrated and are presented below [35] (Table 3). Results that were *discordant* or *complementary* are labelled.

**Table 3:** **Thematic conceptual matrix of integrated qualitative and quantitative findings**

Interview findings	Relationship	Survey findings
Subtheme 1.1: Applicability of recommendations to patient population		
CPGs do not or cannot cater for all patient complexities (n = 25) [1 A]	Agreement	Clinicians agreed that CPGs do not: take patient clinical presentations or complications (74.5%), comorbidities (81.4%) or patient age into account (65.2%) [1B].
CPGs provide a reassuring framework to confirm treatment plans (n = 24) during complex/unfamiliar/rare cases, or new treatments (n = 13) [1 A]	Agreement	CPGs support treatment decision making in complex cases (83.7% agreed) [1B]
CPGS reduce clinical variation and improve patient care (n = 8) [1 A]	Agreement	Clinicians agreed that CPGs help to standardise care (95.4%), and improve patient outcomes (97.7%) [1B]
CPGs are helpful, educational tools, particularly for common cancer cases (n = 9) [1 A]	Agreement	Clinicians agreed that CPGs are convenient sources of advice (97.8%), are practical to use (90.9%), and are good educational tools (100%) [1B]
**Subtheme 1.2: Degree of evidence and level of agreement with evidence underpinning CPGs**		
A lack of agreement with the interpretation of underpinning evidence (particularly when controversial or conflicting) makes it difficult to decide which CPG to follow (n = 6) [2 A]	Discordant	Clinicians disagreed that; their lack of confidence in the interpretation of evidence underpinning CPGs (76.8%), and having multiple CPGs that provide contradictory advice were barriers to adherence (74.4%) [2B]
**Subtheme 1.3: Format- ease of use, references to evidence, and inclusion of patient resources**		
Provision of a summary of evidence with reference to the clinical trials underpinning recommendations was a perceived facilitator (n = 15) [3 A]	Agreement	Clear reference to evidence justifying recommendations facilitates adherence (97.6% agreed) [3B]
Statements that highlight the level of evidence (or consensus) that recommendations are based on **(n = 5)** were considered a facilitator [3 A]	Agreement	Clear labelling of consensus-based recommendations facilitates adherence (97.7% agreed) [3B]
It is a perceived barrier to adherence when CPGs are difficult to navigate (n = 3) [3 A]	Complementary	CPGs are cumbersome and inconvenient (77.3% disagreed) [3B]
**Subtheme 1.4: How up-to-date CPGs are**		
CPGs being slow to be updated (n = 23) [4 A] or underpinned by rapidly changing evidence (n = 19) [2 A] were barriers, while regular updates facilitated adherence (n = 16) [4 A]	Agreement Complementary	Regular updates to CPGs facilitate adherence (100% agreed). CPGs are too out-of-date to be practically useful (68.2% disagreed) [4B]
**Subtheme 1.5: Prescriptiveness of CPG recommendations**		
CPG content that was too broad for complex cases (n = 11) or too rigid, not taking account of emerging evidence (n = 5) were perceived barriers [5 A]	Complementary	CPGs are too rigid to apply to individual patients (63.6% disagreed) [5B]
**Subtheme 2.1: Clinician personality, and the impact of CPGs on autonomy**		
Clinician equipoise and hubris was seen to act as a barrier to adherence (n = 11), as was concern that CPGs can lead to cookbook, or “cookie cutter” medicine, reducing clinician autonomy (n = 2) [6 A]	Discordant	Clinicians disagreed that they; prefer to use their own judgement to inform treatment decisions (74.4%), prefer to continue their routines rather than to change based on CPGs (100%), that CPGs interfere with professional autonomy (93.1%), and that CPGs are too prescriptive (77.3%) [6B]
**Subtheme 2.2: Generational and disciplinary differences in perceptions towards CPGs**		
Some clinicians were perceived as biased by a preference for their discipline, or financially incentivised to complete treatment with the patient rather than engage in multidisciplinary care (n = 7) [7 A]	Discordant	There was no significant difference in CPG attitude scores across subgroups: age groups (p = 0.143), disciplinary groups (p = 0.052), position, (p = 0.307), year clinician graduated from medicine (p = 0.056), or year clinician graduated from oncology specialty (p = 0.592) [7B]
Senior clinicians were perceived as less inclined to refer to CPGs, compared to more junior clinicians (n = 7) [7 A]	Agreement	The age of clinicians (p = 0.007) and the number of MDTs clinicians attend (p = 0.03) were associated with frequency of referral to CPGs [7B]
**Subtheme 2.3: Litigation concerns**		
Concerns around litigation may be a reason CPGs are not developed, particularly regarding treatment doses (n = 1) [8 A]	Discordant	Publishing CPGs increases the risk of malpractice liability (90.9% disagreed) [8B]
Possible litigation and the need to justify and communicate treatment decisions clearly was a facilitator (n = 18) [8 A]	Agreement	Adhering to CPG recommendations covers clinicians medicolegally (79.1% agreed) [8B]
**Subtheme 2.4: Patient age, comorbidities, preferences and logistics**		
Patient preference was a perceived barrier to adherence, including concern about side effects, toxicity, and treatment tolerability (n = 21) [9 A]	Agreement	Patients refusing CPG adherent care is a barrier to adherence (86% agreed) [9B]
Geographic challenges and logistics for rural and remote patients travelling long distances to access treatments was a perceived barrier (n = 10) [9 A]	Discordant	Patient logistics such as living remotely and requiring travel to access treatments acted as a barrier (55.8% disagreed) [9B]
Clinician concern about toxicity or potential side effects of a treatment and treatment tolerability was perceived as a barrier to adherence (n = 7) [9 A]	Discordant	Concern about CPG recommended treatment side effects was a barrier to adherence (74.5% disagreed) [9B]
**Subtheme 3.1: Access to, awareness of and availability of CPGs**		
Some clinicians felt others’ limited awareness of CPGs or where to access them acted as a barrier to adherence (n = 5) [10 A]	Complementary	Clinicians were familiar with the CPGs in their field (97.7%) and felt they were readily available (93.2%). Clinicians disagreed that CPGs were not accessible (76.8%) and that their own lack of awareness of CPG recommendations was a barrier (90.7%)[10B]
Hard to access CPGs that require a login were barriers to adherence (n = 10), and that easy access to CPGs was a facilitator (n = 19) [10 A]	Agreement	Easy access to CPGs with no login requirements facilitates adherence (93% agreed) [10B]
**Subtheme 4.1: Access to treatments recommended by CPGs, resource availability and clinician time**		
Limited availability of drugs (with PBS funding) was a barrier (n = 19) [11 A]	Neutral	A lack of access to CPG recommended drugs was a barrier (50% agreed) [11B]
Organisational support and resources were a facilitator (n = 6) [11 A]	Complementary	There is sufficient support and resources to implement CPGs (50% agreed) [11B]
High clinician workload, limited staffing, and a lack of clinician time can prevent clinicians from looking up CPG recommendations (n = 7) [11 A]	Agreement	Clinicians agreed they do not have time to stay informed about available CPGs (70.5%) and there are so many CPGs available that it is nearly impossible to keep up (63.6%) [11B]
**Subtheme 4.2: A culture of peer review or multidisciplinary review of treatment plans**		
Peer expectation to adhere, fear of looking negligent if non-adherent and knowing that peers adhere were seen as facilitators (n = 10) [12 A]	Agreement	If colleagues’ practice is adherent that encourages clinicians to adhere (76.7% agreed). Clinicians disagreed that they are not expected to use CPGs in their practice setting (97.8%) [12B]
Multidisciplinary engagement or MDT attendance (n = 24) and peer review of treatment decisions (n = 15) [12 A] were seen as facilitators of adherence	Agreement	Clinicians agreed that multidisciplinary review of treatment decisions (93.1%) and peer review of treatment decisions (95.3%) facilitate adherence [12B]. The number of MDTs that clinicians attend was associated with frequency of referring to CPGs (routinely/occasionally vs. rarely/never): 57.9% of clinicians (n = 11) who attend one or two MDTs reported that they routinely or occasionally refer to CPGs, compared to 90.9% of clinicians (n = 20) who attend 3 or more MDTs, p = 0.03 [12B]
**Subtheme 4.3: Referral pathways**		
Patient referral pathways that circumvent multidisciplinary review act as barriers (n = 8) as does lack of awareness by GPs (and patients) of the importance of multidisciplinary review (n = 6) [13 A]	Silence (Expected)	
**Subtheme 5.1: Development, adaptations, and review of CPGs, by an expert development committee**		
Clinicians felt biased CPGs were a barrier to adherence (n = 11) [14 A]	Agreement	Clinicians agreed that adherence was facilitated when CPGs were based on unbiased synthesis of robust scientific evidence (81.8%) or unbiased syntheses of expert opinion (75.1%) [14B]
CPG development by trusted and respected experts transparently and methodically, with multidisciplinary and patient representation on the development committee was a perceived facilitated (n = 16) [14 A]	Complementary	Clinicians agreed that development of CPGs by a trusted expert committee facilitates adherence (90.7%), however 86% disagreed that their lack of confidence in CPG developers was a barrier to adherence [14B]
**Subtheme 5.2: CPG dissemination and implementation strategies**		
Clinical audits of adherence rates were seen as facilitators by some (n = 9) while other clinicians felt audits do not reflect warranted variation, highlighting that low adherence may reflect a poor-quality CPG (n = 11) [15 A].	Agreement	Clinical practice audits of CPG adherence facilitate adherence (88.1% agreed) [15B]
**Subtheme 5.3: Suggested development and implementation improvements**		
Clinicians suggested adapting international CPGs to local needs (n = 9) and developing a comprehensive, continuously updated, dynamic, wiki-like CPGs database, managed by a well-resourced national group (n = 6) [16 A]	Agreement	Clinicians suggested more: frequent updates (n = 4), accessibility (n = 4), development of living (wiki) CPGs (n = 4), reference to CPGs in MDTs (n = 3), development of Australian CPGs or adaptations that reflect PBS drug availability (n = 2) with a template-summary of evidence levels underpinning recommendations (n = 2), and development by a national group (n = 1) [16B].

### [Findings 1AB, 2AB, 3AB, 4AB, 5AB]

Clinicians considered CPGs to be helpful, educational tools that are reassuring frameworks for supporting treatment decisions. They were perceived to reduce clinical variation and improve patient care, while simultaneously being unable to cater for patient complexities. Facilitators included regular CPG updates, and inclusion of a summary of evidence that justifies a recommendation and highlights the level of underpinning evidence. Barriers included a lack of agreement with the CPG interpretation of evidence (*discordant*) and CPGs being difficult to navigate or too rigid (*complementary*).

[Findings 6AB, 7AB, 8AB, 9AB]

Patient preference was a barrier to CPG adherence and potential litigation a facilitator. Younger clinicians and those who attended more MDTs referred to CPGs more often. Barriers such as other clinicians’ hubris; equipoise; and disciplinary preferences; plus concern about CPG-recommended treatment side effect; access challenges for rural patients; and concerns that publishing CPGs increased liability, were *discordant* across the studies.

### [Findings 10AB]

Easy access to CPGs was a facilitator, while concern that other clinicians’ limited awareness of CPGs was a barrier; however, almost all surveyed clinicians reported being familiar with, and having access to, CPGs.

### [Findings 11AB, 12AB]

Peer expectations to adhere to CPGs, multidisciplinary engagement and peer review of treatment decisions were facilitators, while lack of clinician time was a barrier. Surveyed clinicians were divided over whether: limited access to CPG-recommended drugs was a barrier; and if there was enough support and resources to implement CPGs (*complementary*).

### [Findings 14AB, 15AB, 16AB]

Clinical audits, CPGs being based on unbiased synthesis of evidence or expert opinion and being developed by trusted organisations were facilitators. Adapting or tailoring international CPGs to meet local Australian needs and developing living CPGs, managed by a centralised national group, were proposed improvements.

The majority of these findings are in agreement across the two studies, and many have been previously recognised as barriers and facilitators to cancer CPG adherence in the literature [14]. The discordant findings, however, warrant further exploration. Further research is needed to understand treatment access barriers for patients living rurally (who are less likely to receive CPG adherent care than those in metropolitan areas [39, 40]) and the impact on adherence [41]. Similarly, limited access to international CPG-recommended drugs that lack Pharmaceutical Benefits Scheme (PBS) subsidisation and the impact on adherence warrants investigation: universal PBS insurance ensures affordable cancer care by subsidising approved drugs [42]. Further understanding of the impact of organisational support on CPG adherence in Australia is also needed, given organisational support is an established determinant of CPG adherence [43] [21, 44].

## Limitations

The purpose of this study was to assess the frequency of previously published qualitative findings by surveying a broader population of oncology specialists [45]. The sample was dominated by clinicians in NSW, limiting the generalisability of findings. Results from this study should be interpreted with caution, as associations may have been over- or under-estimated [46], the full survey was not validated, and participant self-selection [47] may influence findings. Response rates were smaller than anticipated, despite using recommended strategies (postal surveys, incentives, follow-up) to increase participation [48], potentially reflecting COVID-19-related clinician busyness or burnout [26]. The extended recruitment period may potentially influence findings, although no data discrepancies were noted across the recruitment waves. The small sample size limited the study’s power to statistically compare characteristics and CPG attitude scores across clinician subgroups. Low response rates are common in clinician surveys [48–50], reducing the power to meaningfully compare use of CPGs across respondent subgroups. The comparison of means (e.g., mean CPG attitude scores) generally requires substantially smaller sample sizes, therefore validated scales should be incorporated into surveys, wherever possible, to assess differences between subgroups. In eTable 1 (Supplementary file) we provide means and standard deviations of mean CPG-attitude scores to enable estimation of sample sizes required to detect differences in attitude scores between sub-groups.

The qualitative data that was quantified [33] in the interview study [17] was not validated by a second reviewer [33], limiting its reliability and comparability, and potentially weakening the integration of the data sets [51]. Given the discordant findings identified, a follow-on confirmatory study with a larger sample size, and more nuanced questions, is needed to establish a clearer and more in depth understanding of the determinants of cancer treatment CPG adherence in Australia.

This study has characterised key determinants of cancer treatment CPG adherence in Australia. These findings are intended to inform the development CPG implementation recommendations and strategies to mitigate barriers and utilise facilitators of adherence.

## Electronic supplementary material

Below is the link to the electronic supplementary material.

Supplementary Material 1

## Data Availability

The datasets generated and/or analysed during the current study are not publicly available due to ethics approval data confidentiality requirements, but are available (with restrictions) from the corresponding author on reasonable request and with permission of the South-Western Sydney Local Health District HREC and Macquarie University HREC.

## List of Abbreviations

COSA: Clinical Oncology Society of Australia
COVID-19: Coronavirus disease
CPG: Clinical Practice Guidelines
HREC: Human Research Ethics Committee
MDTs: Multidisciplinary team
MO: Medical Oncologists
NSW: New South Wales
PBS: Pharmaceutical Benefits Scheme
RO: Radiation Oncologist
