# Supplementary material for: Clinician attitudes towards cancer treatment guidelines in Australia
Source: BMC Res Notes. 2023 May 16;16:80. doi: 10.1186/s13104-023-06356-5 (PMC10188226; doi:10.1186/s13104-023-06356-5)
Supplement: Supplementary file 1 — Supplementary Material 1 [file 13104_2023_6356_MOESM1_ESM.docx]

**Supplementary file**

**eTable1. Mean CPG attitude scores across clinician subgroups.**

| **Clinician demographics and practice characteristics** | | **N** | **Mean** | **SD** | **p-value** |
| --- | --- | --- | --- | --- | --- |
| Age | 20-49y | 22 | 41.0 | 7.38 | 0.14 |
|  | 50-79y | 22 | 44.2 | 7.11 |  |
| State of practice | NSW | 37 | 42.1 | 7.35 | 0.35 |
|  | Other | 7 | 45.0 | 7.39 |  |
| LHD | SESLHD | 13 | 39.5 | 4.48 | 0.37 |
|  | SWSLHD | 8 | 44.4 | 6.59 |  |
|  | WSLHD | 10 | 44.1 | 10.5 |  |
|  | Other | 6 | 41.7 | 6.89 |  |
| Specialty | MO | 14 | 39.7 | 5.53 | 0.052 |
|  | RO | 9 | 45.0 | 6.48 |  |
|  | Surgery | 15 | 44.1 | 7.04 |  |
|  | Haematology | 3 | 35.0 | 10.6 |  |
|  | Other | 3 | 48.7 | 9.61 |  |
| Position | Staff Specialist | 28 | 43.1 | 8.24 | 0.31 |
|  | Visiting Medical Officer | 11 | 43.4 | 5.24 |  |
|  | Other | 5 | 37.8 | 4.66 |  |
| Year graduated medicine | 1999 or earlier | 30 | 44.0 | 6.54 | 0.07 |
|  | 2000 or later | 14 | 39.6 | 8.35 |  |
| Year completed oncology specialty | 1999 or earlier | 14 | 43.3 | 6.62 | 0.74 |
|  | 2000 or later | 29 | 42.5 | 7.80 |  |
| Country graduated medicine in | Australia | 36 | 42.2 | 6.80 | 0.49 |
|  | Other | 8 | 44.3 | 9.85 |  |
| Country where oncology specialty completed | Australia | 40 | 42.3 | 7.16 | 0.25 |
|  | UK | 2 | 51.0 | 11.3 |  |
|  | Australia and UK/CA | 2 | 40.5 | 6.36 |  |
| Proportion of clinical practice in public settings | <50% | 16 | 41.4 | 8.68 | 0.41 |
|  | 50% or more | 28 | 43.3 | 6.54 |  |
| Proportion of clinical practice in metropolitan settings | 50-74% | 2 | 41.5 | 13.4 | 0.83 |
|  | 75%> | 42 | 42.6 | 7.22 |  |
| Number of cancer streams clinicians specialise in | 1 | 20 | 41.4 | 7.31 | 0.053 |
|  | 2-3 cancer streams | 11 | 40.1 | 6.91 |  |
|  | 4 or more cancer streams | 13 | 46.6 | 6.59 |  |
| MDT membership | No | 1 | 31.0 | - | 0.11 |
|  | Yes | 43 | 42.9 | 7.21 |  |
| Number of MDT memberships | 1 or 2 MDTs | 18 | 42.1 | 5.40 | 0.64 |
|  | 3 or more MDTs | 21 | 43.1 | 8.31 |  |
| Frequency of MDT attendance | More than once per week | 27 | 43.9 | 7.37 | 0.23 |
|  | Weekly or less often | 16 | 41.1 | 6.82 |  |
| Frequency clinicians refer to CPGs | More than once per week | 33 | 42.9 | 7.72 | 0.59 |
|  | Weekly or less often | 11 | 41.6 | 6.31 |  |
| Frequency practice is adherent with CPG recommendations | Routinely | 32 | 42.8 | 7.07 | 0.71 |
|  | Occasionally/more 50% | 12 | 41.9 | 8.35 |  |
